# Supplementary material for: Long-Term Aberrations To Cerebellar Endocannabinoids Induced By Early-Life Stress
Source: Sci Rep. 2020 Apr 29;10:7236. doi: 10.1038/s41598-020-64075-4 (PMC7190863; doi:10.1038/s41598-020-64075-4)
Supplement: Supplementary file 1 — Supplementary information. [file 41598_2020_64075_MOESM1_ESM.docx]

**SUPPLEMENTARY INFORMATION**

**for**

**LONG-TERM ABERRATIONS TO CEREBELLAR ENDOCANNABINOIDS INDUCED BY EARLY-LIFE STRESS**

**Authors**: Alexandra B. Moussa-Tooks, B.A.^1,2^, Eric R. Larson^1^, Alex F. Gimeno^1^, Emma Leishman, Ph.D.^1,2^, Lisa A. Bartolomeo, B.S.^1^, Heather B. Bradshaw, Ph.D.^1,2^, John T. Green, Ph.D.^3^, Brian F. O’Donnell, Ph.D.^1,2,4^, Ken Mackie, M.D.^1,2,5^, William P. Hetrick, Ph.D.^1,2,4^

^1^Psychological and Brain Sciences, Indiana University, Bloomington, IN

^2^Program in Neuroscience, Indiana University, Bloomington, IN

^3^Department of Psychological Science, University of Vermont, Burlington, VT

^4^Department of Psychiatry, Indiana University School of Medicine, Indianapolis, IN

^5^Linda and Jack Gill Center for Biomolecular Science, Indiana University, Bloomington, IN

**Running Title:** Early-life Stress and Cerebellar Endocannabinoids

**Corresponding Author:** William P. Hetrick

1101 E. 10^th^ St.

Bloomington, IN

47405

Phone: (812) 855-2620

Fax: (812) 855-4691

Email: whetrick@indiana.edu

*Key Words: limited nesting, cerebellum, early-life stress, endocannabinoids, vulnerability, sex-differences*

| **Supplementary Table 1: Crus/HVI Endocannabinoids** | | | | | |
| --- | --- | --- | --- | --- | --- |
| **Lipid Species** | **Male** | | **Female** | |  |
|  | **Normal**  **Rearing**  (N=8) | **Limited**  **Bedding**  (N=8) | **Normal**  **Rearing**  (N=6) | **Limited**  **Bedding**  (N=7) | ***p*-value*** |
| ***N*-acyl ethanolamine** |  |  |  |  |  |
| *N*-palmitoyl ethanolamine | 4.9E-10±  6.25E-11 | 5.52E-10±  2.86E-11 | 3.77E-10±  5.46E-11 | 4.64E-10±  5.94E-11 | NS |
| *N*-stearoyl ethanolamine | *2.41E-10±*  *3.97E-11* | 2.34E-10±  2.83E-11 | *1.21E-10±*  *2.52E-11* | 2.11E-10±  4.03E-11 | *0.028* |
| *N*-oleoyl ethanolamine | *2.1E-09±*  *3.28E-10* | 1.96E-09±  3.31E-10 | *1.03E-09±*  *2.09E-10* | 1.66E-09±  3.17E-10 | *0.027* |
| *N*-linoleoyl ethanolamine | *2.01E-11±*  *1.93E-12* | 1.72E-11±  1.58E-12 | *9.66E-12±*  *1.24E-12* | 1.45E-11±  1.78E-12 | *<0.001* |
| *N*-arachidonoyl ethanolamine | 1.79E-11±  2.91E-12 | 1.25E-11±  5.37E-13 | 1.58E-11±  2.34E-12 | 1.62E-11±  2.59E-12 | NS |
| *N*-docosahexaenoyl ethanolamine | 3.16E-11±  2.66E-12 | 3.06E-11±  9.74E-13 | 3.01E-11±  4.28E-12 | 3.04E-11±  4.26E-12 | NS |
| ***N*-acyl glycine** |  |  |  |  |  |
| *N*-palmitoyl glycine | *4.85E-11±*  *3.99E-12* | **4.68E-11±**  **3.65E-12** | *6.46E-11±*  *7.94E-12* | **6.09E-11±**  **2.64E-12** | *0.021*  **0.045** |
| *N*-stearoyl glycine | 1..45E-11±  9.22E-13 | 1.4E-11±  9.11E-13 | 1.5E-11±  1.19E-12 | 1.64E-11±  1.42E-12 | NS |
| *N*-oleoyl glycine | 4.35E-12±  5.74E-13 | 3.6E-12±  2.47E-13 | 3.19E-12±  3.06E-13 | 4.26E-12±  4.51E-13 | NS |
| *N*-linoleoyl glycine | 6.95E-13±  8.63E-14 | 8E-13±  1.73E-13 | 8.49E-13±  2.43E-13 | 7.63E-13±  1.39E-13 | NS |
| *N*-arachidonoyl glycine | 7.09E-12±  2.22E-13 | 5.83E-12±  8.92E-14 | 6.48E-12±  8.35E-13 | 6.23E-12±  7.1E-13 | NS |
| *N*-docosahexaenoyl glycine | *1.5E-12±*  *1.22E-13* | 1.61E-12±  3.09E-13 | *2.08E-12±*  *8.27E-14*♀ | 1.46E-12±  1.16E-13♀ | *0.005*  0.004♀ |
| ***N*-acyl taurine** |  |  |  |  |  |
| *N*-palmitoyl taurine | 1.25E-10±  8.87E-12 | 1.16E-10±  8.16E-12 | 1.22E-10±  3.31E-12 | 1.26E-10±  6.23E-12 | NS |
| *N*-stearoyl taurine | 1.08E-10±  7.84E-12 | 1.26E-10±  9.89E-12 | 1.34E-10±  1.32E-11 | 1.16E-10±  6.49E-12 | NS |
| *N*-oleoyl taurine | 2.55E-11±  2.25E-12 | 2.04E-11±  2.33E-12 | 2.2E-11±  3.83E-12 | 2.17E-11±  2.48E-12 | NS |
| *N*-arachidonoyl taurine | 2.05E-10±  1.65E-11 | 2.22E-10±  1.6E-11 | 2.06E-10±  1.61E-11 | 2.05E-10±  1.15E-11 | NS |
| **2-acyl glycerol** |  |  |  |  |  |
| 2-palmitoyl glycerol | 3.37E-09±  5.2E-10 | 4.41E-09±  1.04E-09 | 4.69E-09±  1.28E-09 | 2.91E-09±  4.66E-10 | NS |
| 2-oleoyl glycerol | 6.36E-09±  6.41E-10 | 6.29E-09±  5.2E-10 | 5.89E-09±  6.02E-10 | 4.88E-09±  5.23E-10 | NS |
| 2-linoleoyl glycerol | 6.48E-10±  3.42E-11 | **7.01E-10±**  **6.9E-11** | 6.31E-10±  8.07E-11 | **4.96E-10±**  **5.14E-11** | **0.020** |
| 2-arachidonoyl glycerol | *1.22E-09±*  *3.6E-11* | 1.29E-09±  6.91E-11 | *1.26E-09±*  *5.1E-11♀* | 1.09E-09±  1.18E-10♀ | *0.020*  0.002♀ |
| **Free Fatty Acids** |  |  |  |  |  |
| Oleic acid | 3.84E-10±  4.09E-11 | 4.13E-10±  3.95E-11 | 3.23E-10±  3.32E-11 | 3.33E-10±  4.55E-11 | NS |
| Linoleic acid | 6.07E-10±  4.26E-11 | **6.25E-10±**  **2.93E-11** | 5.11E-10±  6.17E-11 | **4.87E-10±**  **5.66E-11** | **0.047** |
| Arachidonic acid | 7.23E-09±  4.26E-10 | 8E-09±  4.43E-10 | 7.65E-09±  5.65E-10 | 7.48E-09±  6.51E-10 | NS |
| **Prostaglandins** |  |  |  |  |  |
| PGE_2_ | 9.54E-11±  7.62E-12 | 8.29E-11±  9.18E-12 | 8.18E-11±  6.88E-12 | 6.82E-11±  6.51E-12 | NS |
| PGF_2α_ | *1.66E-10±*  *1.34E-11* | 2.04E-10±  7.92E-12 | *2.25E-10±*  *1.81E-11* | 1.96E-10±  2.33E-11 | *0.017* |
| 6-ketoPGF_1α_ | BAL | BAL | BAL | BAL | -- |
| **Sample Mass** |  |  |  |  |  |
| Sample Mass | *0.02±1.12E-3* | 0.02±7.3E-4 | *0.02±2.1E-3* | 0.02±8.1E-4 | *0.001* |
| Data are moles per gram tissue and are shown as means ± SE. Values in light face have no significant difference among the groups. *Least Significant Difference corrected *p*≤0.05. *Italicized* values denote a significant sex effect for normally reared animals, whereas those in **bold** denote a significant sex effect for limited bedding animals; ♀=significant treatment effect for females, NS=Not Significant*.* BAL=Below Analytical Limits. | | | | | |

| **Supplementary Table 2: Interpositus Nucleus Endocannabinoids** | | | | | |
| --- | --- | --- | --- | --- | --- |
| **Lipid Species** | **Male** | | **Female** | |  |
|  | **Normal**  **Rearing**  (N=8) | **Limited**  **Bedding**  (N=8) | **Normal**  **Rearing**  (N=6) | **Limited**  **Bedding**  (N=7) | ***p*-value*** |
| ***N*-acyl ethanolamine** |  |  |  |  |  |
| *N*-palmitoyl ethanolamine | 2.81E-10±  3.18E-11 | 3.29E-10±  3.87E-11 | 3.13E-10±  4.37E-11 | 3.46E-10±  3.11E-11 | NS |
| *N*-stearoyl ethanolamine | 7.45E-11±  1.14E-11 | 7.31E-11±  7.65E-12 | 7.17E-11±  5.79E-12 | 9.10E-11±  1.31E-11 | NS |
| *N*-oleoyl ethanolamine | 2.25E-10±  2.86E-11 | 2.24E-10±  2.03E-11 | 2.10E-10±  2.37E-11 | 2.35E-10±  2.84E-11 | NS |
| *N*-linoleoyl ethanolamine | *2.93E-11±*  *2.86E-12* | 2.75E-11±  1.85E-12 | *2.03E-11±*  *2.02E-12* | 2.10E-11±  2.71E-12 | *0.020* |
| *N*-arachidonoyl ethanolamine | 1.47E-11±  1.60E-12 | 1.11E-11±  3.84E-13 | 1.10E-11±  1.00E-12 | 1.16E-11±  2.15E-12 | NS |
| *N*-docosahexaenoyl ethanolamine | 2.57E-11±  2.87E-12 | 2.29E-11±  1.79E-12 | 2.08E-11±  2.25E-12 | 2.25E-11±  2.36E-12 | NS |
| ***N*-acyl glycine** |  |  |  |  |  |
| *N*-palmitoyl glycine | 1.39E-11±  1.14E-12 | 1.29E-11±  1.53E-12 | 1.50E-11±  2.88E-12 | 1.81E-11±  1.98E-12 | NS |
| *N*-stearoyl glycine | 2.42E-12±  1.82E-13 | 2.41E-12±  4.29E-13 | 2.39E-12±  6.30E-13 | 3.24E-12±  4.56E-13 | NS |
| *N*-oleoyl glycine | 2.94E-12±  2.31E-13 | **2.47E-12±**  **1.74E-13** | 3.56E-12±  5.58E-13 | **3.65E-12±**  **5.59E-13** | **0.038** |
| *N*-linoleoyl glycine | 9.89E-13±  1.35E-13 | 8.34E-13±  1.65E-13 | 1.26E-12±  3.23E-13 | 1.01E-12±  1.12E-13 | NS |
| *N*-arachidonoyl glycine | 6.71E-12±  9.91E-13 | 5.69E-12±  4.78E-13 | 5.37E-12±  8.62E-13 | 6.62E-12±  1.47E-12 | NS |
| *N*-docosahexaenoyl glycine | 2.55E-12±  2.40E-13 | 2.01E-12±  1.92E-13 | 2.78E-12±  2.76E-13 | 3.47E-12±  5.52E-13 | NS |
| ***N*-acyl taurine** |  |  |  |  |  |
| *N*-palmitoyl taurine | 1.57E-10±  9.45E-12 | 1.51E-10±  7.65E-12 | 1.57E-10±  1.56E-11 | 1.44E-10±  7.70E-12 | NS |
| *N*-stearoyl taurine | 1.72E-10±  4.10E-12 | 1.82E-10±  1.39E-11 | 1.78E-10±  1.24E-11 | 1.89E-10±  1.26E-11 | NS |
| *N*-oleoyl taurine | 2.79E-11±  2.86E-12 | 3.49E-11±  5.21E-12 | 3.04E-11±  3.22E-12 | 3.59E-11±  4.54E-12 | NS |
| *N*-arachidonoyl taurine | *2.53E-11±*  *7.46E-12* | **2.53E-11±**  **7.42E-12** | *1.68E-11±*  *3.42E-12* | **1.59E-11±**  **5.18E-12** | *0.017*  **0.009** |
| **2-acyl glycerol** |  |  |  |  |  |
| 2-palmitoyl glycerol | 2.85E-08±  8.60E-09 | 2.92E-08±  1.11E-08 | 2.56E-08±  1.21E-08 | 4.05E-08±  1.67E-08 | NS |
| 2-oleoyl glycerol | 9.81E-09±  1.44E-09 | 7.79E-09±  9.90E-10 | 8.85E-09±  1.24E-09 | 9.65E-09±  1.62E-09 | NS |
| 2-linoleoyl glycerol | 9.45E-10±  1.14E-10 | 7.13E-10±  5.86E-11 | 7.95E-10±  7.76E-11 | 9.00E-10±  7.54E-11 | NS |
| 2-arachidonoyl glycerol | 1.49E-09±  1.65E-10♂ | **1.07E-09±**  **7.53E-11**♂ | 1.54E-09±  9.19E-11 | **1.80E-09±**  **8.97E-11** | 0.015♂  **<0.001** |
| **Free Fatty Acids** |  |  |  |  |  |
| Oleic acid | 2.69E-10±  2.41E-11 | 2.56E-10±  2.69E-11 | 2.24E-10±  1.51E-11 | 2.62E-10±  3.89E-11 | NS |
| Linoleic acid | *6.04E-13±*  *7.17E-14* | 4.44E-13±  6.41E-14 | *3.35E-13±*  *6.12E-14* | 2.70E-13±  4.90E-14 | *0.008* |
| Arachidonic acid | *4.35E-10±*  *2.05E-11*♂ | 3.58E-10±  2.28E-11♂ | *3.72E-10±*  *1.12E-11* | 3.34E-10±  2.38E-11 | *0.049*  0.011♂ |
| **Prostaglandins** |  |  |  |  |  |
| PGE_2_ | *8.93E-11±*  *6.77E-12* | **7.57E-11±**  **4.02E-12** | *6.56E-11±*  *7.07E-12*♀ | **9.93E-11±**  **9.35E-12**♀ | *0.026*  **0.024**  0.004♀ |
| PGF_2α_ | BAL | BAL | BAL | BAL |  |
| 6-ketoPGF_1α_ | 3.18E-11±  4.66E-12 | 3.72E-11±  4.04E-12 | 4.07E-11±  6.82E-12 | 4.79E-11±  7.83E-12 | NS |
| **Sample Mass** |  |  |  |  |  |
| Sample Mass | *0.02±5.03E-4* | **0.02±6.34E-4** | *0.02±9.30E-4* | **0.02±1.02E-3** | *0.020*  **0.001** |
| Data are moles per gram tissue and are shown as means ± SE. Values in light face have no significant difference among the groups. *Least Significant Difference corrected *p*≤0.05. *Italicized* values denote a significant sex effect for normally reared animals, whereas those in **bold** denote a significant sex effect for limited bedding animals; ♂️=significant treatment effect for males, ♀=significant treatment effect for females, NS=Not Significant*.* BAL=Below Analytical Limits. | | | | | |

| **Supplementary Table 3: Dorsal Hippocampus Endocannabinoids** | | | | | |
| --- | --- | --- | --- | --- | --- |
| **Lipid Species** | **Male** | | **Female** | |  |
|  | **Normal**  **Rearing**  (N=8) | **Limited**  **Bedding**  (N=8) | **Normal**  **Rearing**  (N=6) | **Limited**  **Bedding**  (N=7) | ***p*-value*** |
| ***N*-acyl ethanolamine** |  |  |  |  |  |
| *N*-palmitoyl ethanolamine | 5.62E-10±  7.69E-11 | 6.47E-10±  1.16E-10 | 3.55E-10±  4.5E-11 | 4.12E-10±  8.05E-11 | NS |
| *N*-stearoyl ethanolamine | 5.2E-11±  1.07E-11 | 7.91E-11±  9.59E-12 | 4.53E-11±  8.46E-12 | 5.16E-11±  1.16E-11 | NS |
| *N*-oleoyl ethanolamine | 6.36E-10±  4.76E-11 | 7.61E-10±  7.31E-11 | 4.73E-10±  3.82E-11 | 6.39E-10±  1.3E-10 | NS |
| *N*-linoleoyl ethanolamine | 1.07E-10±  1.29E-11 | 1.04E-10±  1.28E-11 | 8.61E-11±  1.26E-11 | 9.44E-11±  1.28E-11 | NS |
| *N*-arachidonoyl ethanolamine | 4.66E-11±  7.07E-12 | 4.21E-11±  5.11E-12 | 4.15E-11±  8.57E-12 | 4.81E-11±  7.18E-12 | NS |
| *N*-docosahexaenoyl ethanolamine | 3.83E-11±  4.87E-12 | 4.01E-11±  5.47E-12 | 3.1E-11±  5.29E-12 | 4.47E-11±  8.96E-12 | NS |
| ***N*-acyl glycine** |  |  |  |  |  |
| *N*-palmitoyl glycine | 1.15E-11±  6.07E-13 | 1.5E-11±  9.45E-13 | 1.4E-11±  2.27E-12 | 1.29E-11±  1.57E-12 | NS |
| *N*-stearoyl glycine | 6.75E-12±  4.34E-13 | 6.63E-12±  4.92E-13 | 6.85E-12±  5E-13 | 6.97E-12±  3.85E-13 | NS |
| *N*-oleoyl glycine | 5.28E-12±  3.47E-13 | 6.2E-12±  2.66E-13 | 5.57E-12±  3.36E-13 | 6.25E-12±  3.89E-13 | NS |
| *N*-linoleoyl glycine | 4.72E-13±  4.11E-14 | 5.97E-13±  5.08E-14 | 6.15E-13±  8.04E-14 | 5.81E-13±  9.82E-14 | NS |
| *N*-arachidonoyl glycine | 1.15E-11±  1E-12 | 1.15E-11±  8.6E-13 | 1.23E-11±  9.05E-13 | 1.35E-11±  1.65E-12 | NS |
| *N*-docosahexaenoyl glycine | 8.7E-13±  1.15E-13 | 1.02E-12±  1.27E-13 | 8.61E-13±  1.26E-13 | 1.28E-12±  3.05E-13 | NS |
| ***N*-acyl taurine** |  |  |  |  |  |
| *N*-palmitoyl taurine | 1.58E-10±  1.54E-11 | 1.55E-10±  2.04E-11 | 1.37E-10±  9.47E-12 | 1.46E-10±  1.85E-11 | NS |
| *N*-stearoyl taurine | 6.34E-11±  7.14E-12 | 7.23E-11±  6.43E-12 | 7E-11±  8.21E-12 | 7.16E-11±  8.72E-12 | NS |
| *N*-oleoyl taurine | 1.73E-11±  1.97E-12 | 1.85E-11±  2.48E-12 | 1.78E-11±  1.9E-12 | 1.63E-11±  2.27E-12 | NS |
| *N*-arachidonoyl taurine | 3.8E-11±  4.7E-12 | 3.75E-11±  5.66E-12 | 4.43E-11±  3.95E-12 | 3.83E-11±  4.49E-12 | NS |
| **2-acyl glycerol** |  |  |  |  |  |
| 2-palmitoyl glycerol | 6.56E-09±  1.06E-09 | 7.76E-09±  1.15E-09 | 4.65E-09±  7.35E-10 | 5.54E-09±  8.32E-10 | NS |
| 2-oleoyl glycerol | *1.4E-08±*  *2.24E-09* | 1.26E-08±  2.26E-09 | *7.33E-09±*  *1.26E-09* | 8.59E-09±  1.55E-09 | *0.029* |
| 2-linoleoyl glycerol | *9.55E-11±*  *6.97E-12* | **9.31E-11±**  **8.44E-12** | *5.05E-11±*  *4.97E-12* | **4.95E-11±**  **4.94E-12** | *<0.001*  **<0.001** |
| 2-arachidonoyl glycerol | *5.52E-10±*  *7.7E-11* | 4.39E-10±  7.76E-11 | *3.19E-10±*  *6.8E-11* | 3.3E-10±  5.33E-11 | *0.034* |
| **Free Fatty Acids** |  |  |  |  |  |
| Oleic acid | 4.8E-09±  4.75E-10 | 5.52E-09±  5.33E-10 | 4.5E-09±  7.6E-10 | 5.13E-09±  5.51E-10 | NS |
| Linoleic acid | 5.04E-10±  2.96E-11 | 5.63E-10±  4.11E-11 | 4.22E-10±  3.64E-11 | 4.91E-10±  3.59E-11 | NS |
| Arachidonic acid | 3.13E-09±  1.12E-10 | 3.46E-09±  1.87E-10 | 3.49E-09±  3.05E-10 | 3.5E-09±  1.88E-10 | NS |
| **Prostaglandins** |  |  |  |  |  |
| PGE_2_ | 1.98E-11±  1.54E-12♂ | 2.88E-11±  1.82E-12♂ | 2.47E-11±  1.6E-12 | 2.53E-11±  2.34E-12 | 0.001♂ |
| PGF_2α_ | *2.71E-11±*  *1.16E-12*♂ | 3.75E-11±  9.46E-13♂ | *3.21E-11±*  *2.53E-12* | 3.33E-11±  1.41E-12 | <0.001♂  *0.027* |
| 6-ketoPGF_1α_ | BAL | BAL | BAL | BAL | -- |
| **Sample Mass** |  |  |  |  |  |
| Sample Mass | 0.02±7.32E-4 | 0.02±1.24E-3 | 0.02±6.29E-4 | 0.02±6.72E-4 | NS |
| Data are moles per gram tissue and are shown as means ± SE. Values in light face have no significant difference among the groups. *Least Significant Difference corrected *p*≤0.05. *Italicized* values denote a significant sex effect for normally reared animals, whereas those in **bold** denote a significant sex effect for limited bedding animals; ♂=significant treatment effect for males, NS=Not Significant*.* BAL=Below Analytical Limits. | | | | | |
